# Supplementary material for: Cancer associated talin point mutations disorganise cell adhesion and migration
Source: Sci Rep. 2021 Jan 11;11:347. doi: 10.1038/s41598-020-77911-4 (PMC7801617; doi:10.1038/s41598-020-77911-4)
Supplement: Supplementary file 1 — Supplementary Information 1. [file 41598_2020_77911_MOESM1_ESM.docx]

Supplementary information for

**Cancer associated talin point mutations disorganise cell adhesion and migration**

Latifeh Azizi^1^, Alana R. Cowell^2^, Vasyl V. Mykuliak^1^, Benjamin T. Goult^2*^, Paula Turkki^1,3*^, Vesa P. Hytönen^1,3*^

^1^BioMediTech, Faculty of Medicine and Health Technology, Tampere University, Tampere, Finland.

^2^School of Biosciences, University of Kent, Canterbury, Kent, CT2 7NJ, UK.

^3^Fimlab Laboratories, Tampere, Finland.

**Corresponding authors: Benjamin T. Goult: B.T.Goult@kent.ac.uk, Paula Turkki: paula.turkki@tuni.fi, Vesa P. Hytönen: vesa.hytonen@tuni.fi*

**Supplementary Materials and Methods**

**Screening TLN1 mutations using bioinformatics**

In order to classify all mutations into different classes and groups, we used structural information from the protein data bank (PDB): 3IVF ^1^, 4F7G ^2^, 1SJ7 ^3^, 2L7A ^4^, 2X0C ^5^, 2KBB ^6^, 3DYJ ^7^ and 2QDQ ^8^. We examined each mutations position on the talin domains and the location on the structure (surface or buried). This was done using an algorithm developed by our group ^9^ and using PyMOL software to visually observe the position of the mutation. The mutations were classified using PON–P2 tool into pathogenic, neutral and unknown to obtain the probability for pathogenicity. PON–P2 is freely available at http://structure.bmc.lu.se/PON–P2/ ^10^. The BLOSUM 62 matrix ^11^ was used to evaluate the severity of the mutations. The degree of evolutionary conservation of the amino acid in the talin–1 sequence was investigated using ConSurf (https://consurf.tau.ac.il/ ^12^). The amino acid substitution matrices CBSM60 was used to analyse the sequence–structure relationship of the protein ^13^. We also investigated whether the mutation was located in known ligand–binding sites and reported the recurrence and the substitution of the amino acid from hydrophobicity to hydrophilicity and vice versa. The mutations were classified into six group based on their location: surface, buried, on a loop, on a linker but buried, between the helices buried, and on the dimerisation domain. These were given the position code of 0.4, 1, 0.2, 1, 0 and 0.8 respectively. For the change in polarity, we gave a score

of one when the amino acid was mutated from hydrophilic residue to hydrophobic residue or from

hydrophobic residue to hydrophilic residue. Considering all these variables, we estimated the scoring coefficient according to the importance feature of each factor using formula n_1_X1+n_2_X2+…+n_n_Xn (n is the scoring coefficient and X is the variable). We tried multiple scoring factor combinations to obtain mutations appearing most frequently with a higher score. Finally, we generated a table from which we selected ten mutants predicted to represent the most drastic mutations based on “total score”. The scoring coefficient to calculate the “total score” of one iteration in Table 1 is as follows: 2*(location within the subdomain code) + 0*(ligand binding code) + 4*(ConSurf code) + 0*(BLOSUM 62) + 2*(PON–P2) 1* (CBSM60) + 0.05*(polarity change from hydrophilic to hydrophobic) + 0.4*(polarity change from hydrophobic to hydrophilic). Altogether, nine iterations were done.

**Prediction of the deleterious effect of mutation**

We normalised the score for each investigated amino–acid substitution between zero and one, with one being the most deleterious. Considering all these factors, we calculated a final score using equation n_1_X1+n_2_X2+…+n_n_Xn by giving different indexes (scoring coefficient, n) a range of zero to five, where a higher value indicated a greater effect of the variant. Using the variables (X), “location within the subdomain”, “ligand binding”, “ConSurf”, “BLOSUM62”, “PON–P2”, “CBSM60” and “polarity change” we tested different relative weightings (for example 2, 0, 4, 0, 2, 1, 0.05, 0.4) and ran nine iterations. Each time, we pooled the top 10 mutations which had the highest score. Based on these criteria, 78 mutants received a score above five, and 10 mutations with the highest scores (>7) likely to be detrimental were taken forward for further analysis.

**Antibodies**

**Table S1:** Antibodies used in this study. Antibodies were diluted in 1.5% BSA, 0.1% Triton–X /PBS buffer. Appropriate secondary antibodies from LI–CORE and a LI–CORE imaging system was used.

| **Antibody** | **Manufacturer** | **Method** | **Dilution used** |
| --- | --- | --- | --- |
| anti–vinculin | Merck, clone hVIN, V9131, RRID:AB_477629 | Immunostaining/  western blot | 1:100 / 1:1000 |
| anti–FAK–pY397 | Abcam, ab81298 [EP2160Y], RRID:AB_1640500 | Immunostaining/  western blot | 1:100 / 1:1000 |
| FAK (clone 77) | BD Biosciences, 610088 | western blot | 1:1000 |
| DLC–1 (H–260) | Santa Cruz Biotechnology, sc–32931 | Immunostaining | 1:100 |
| Integrin β1–chain CD29, clone 9EG7 | BD Pharmingen (Cat:553715) | Immunostaining | 1:200 |
| anti–paxillin | BD Biosciences, 349/Paxillin, 610051, RRID:AB_397463 | Immunostaining/  western blot | 1:100 / 1:1000 |
| GFP antibody | Sicgen AB0020–200 | western blot | 1:1000 |
| Actin | Millipore, MAB 1501R, RRID: AB_2223041 | western blot | 1:2000 |
| Alexa Fluor 568 phalloidin | Life Technologies | Immunostaining | 1:40 |
| Alexa Fluor 568 goat anti–rabbit IgG | Life Technologies A11011 | Immunostaining | 1:200 |
| Alexa Fluor 568 goat anti–mouse IgG | Molecular probes, A11004 | Immunostaining | 1:200 |

**Image analyses**

**Protein expression level and co–localisation quantification from confocal images.** For the quantification of expression level of proteins, the total intensity signal was determined from transfected cells. For the co–localisation quantification of protein intensity, 10–15 adhesion sites per cell were selected based on the EGFP–talin channel using circular selection (0.7 µm) of ImageJ and selection was copied to the red fluorescence channel. Background was assessed from the EGFP channel using circular selections (2.2 µm) from areas devoid of EGFP signal and these areas were again copied to the red fluorescence channel. Protein expression and co–localisation was measured using ImageJ.

**Analyses of adhesion size and number**. To determine the adhesion size and number in transfected cells, we conducted particle analysis using ImageJ particle analyser. First, the threshold range was set to clear out the background noise. Then we selected areas from cell boundaries (roughly one third of the cell membrane) and analysed the adhesions sites based on the signal from the EGFP channel. Cut–off sizes of < 0.1 and > 20 µm^2^ were used in the analyses. Results are shown as ratio of adhesion area and as the number of individual adhesions sites per total selected area per.

**Supplementary** **References**

1. Elliott, P. R. *et al*. The Structure of the talin head reveals a novel extended conformation of the FERM domain. *Structure* 18, 1289-1299 (2010).

2. Song, X. *et al*. A novel membrane-dependent on/off switch mechanism of talin FERM domain at sites of cell adhesion. *Cell Res.* 22, 1533-1545 (2012).

3. Papagrigoriou, E. *et al*. Activation of a vinculin-binding site in the talin rod involves rearrangement of a five-helix bundle. *EMBO J.* 23, 2942-2951 (2004).

4. Goult, B. T. *et al*. RIAM and vinculin binding to talin are mutually exclusive and regulate adhesion assembly and turnover. *J. Biol. Chem.* 288, 8238-8249 (2013).

5. Gingras, A. R. *et al*. Central region of talin has a unique fold that binds vinculin and actin. *J. Biol. Chem.* 285, 29577-29587 (2010).

6. Goult, B. T. *et al*. The structure of an interdomain complex that regulates talin activity. *J. Biol. Chem.* 284, 15097-15106 (2009).

7. Gingras, A. R. *et al*. Structural determinants of integrin binding to the talin rod. *J. Biol. Chem.* 284, 8866-8876 (2009).

8. Gingras, A. R. *et al*. The structure of the C-terminal actin-binding domain of talin. *EMBO J.* 27, 458-469 (2008).

9. Nurminen, A. & Hytönen, V. P. StructureMapper: a high-throughput algorithm for analyzing protein sequence locations in structural data. *Bioinformatics* 34, 2302-2304 (2018).

10. Niroula, A., Urolagin, S. & Vihinen, M. PON-P2: Prediction Method for Fast and Reliable Identification of Harmful Variants. *PloS one* 10, e0117380 (2015).

11. Henikoff, S. & Henikoff, J. G. Amino acid substitution matrices from protein blocks. *Proc. Natl. Acad. Sci. U. S. A.* 89, 10915-10919 (1992).

12. Ashkenazy, H. *et al*. ConSurf 2016: an improved methodology to estimate and visualize evolutionary conservation in macromolecules. *Nucleic Acids Res.* 44, 344 (2016).

13. Liu, X. & Zheng, W. M. An amino acid substitution matrix for protein conformation identification. *J. Bioinform Comput. Biol.* 4, 769-782 (2006).
