# Supplementary figures and images for: Cancer associated talin point mutations disorganise cell adhesion and migration
Source: Sci Rep. 2021 Jan 11;11:347. doi: 10.1038/s41598-020-77911-4 (PMC7801617; doi:10.1038/s41598-020-77911-4)

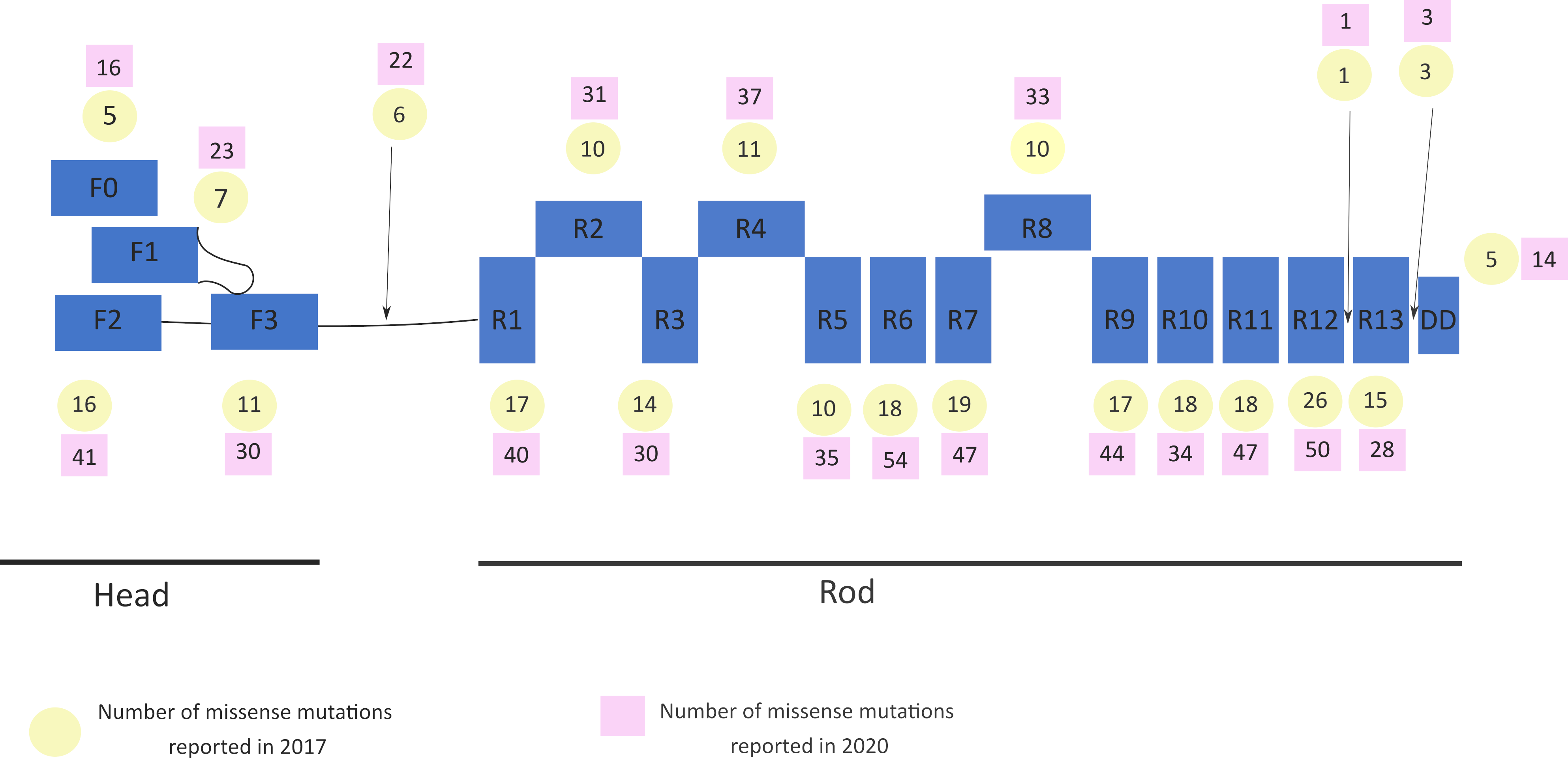

Supplement: Supplementary file 2 — Supplementary Information 2. [file 41598_2020_77911_MOESM2_ESM.tiff]

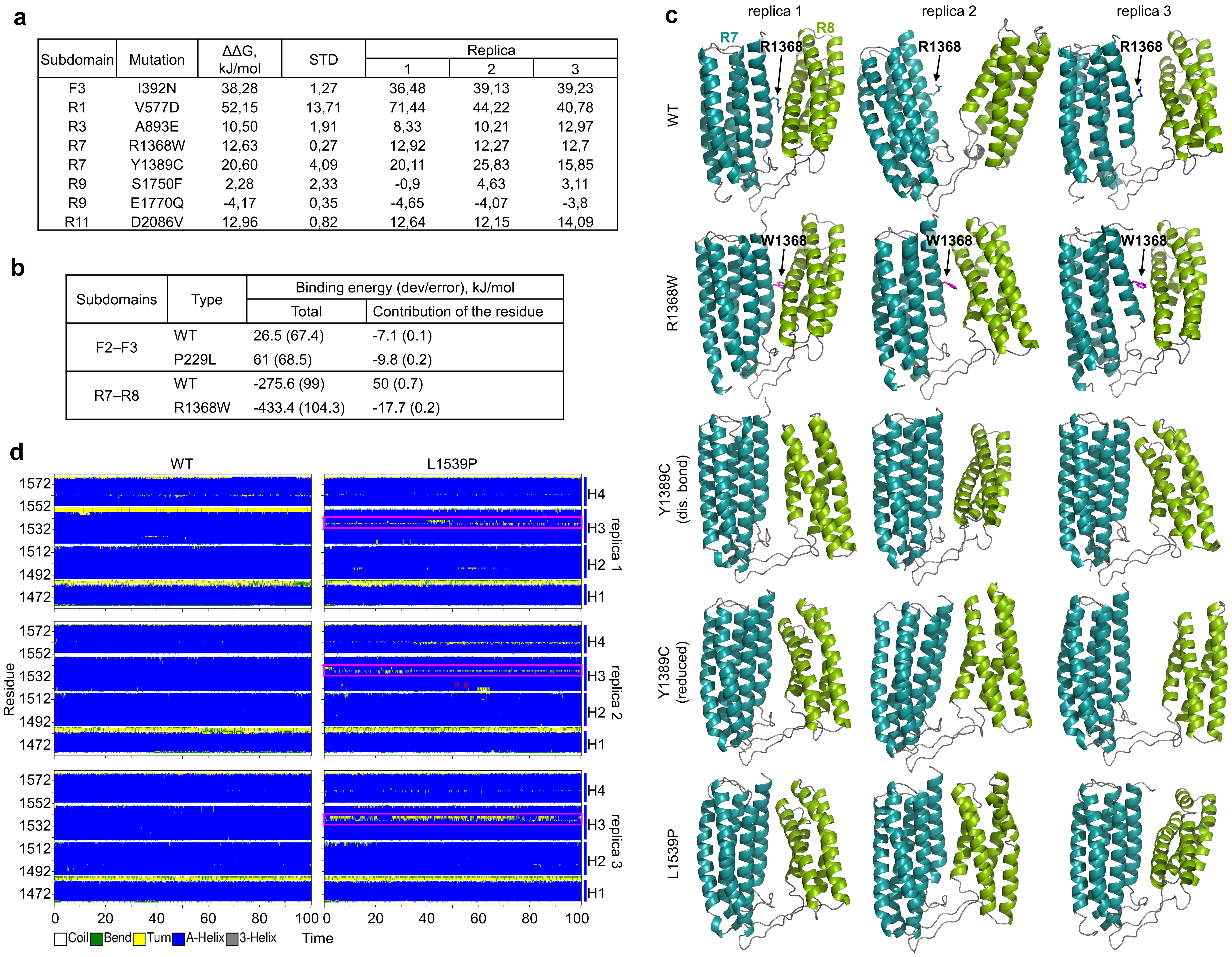

Supplement: Supplementary file 3 — Supplementary Information 3. [file 41598_2020_77911_MOESM3_ESM.tiff]

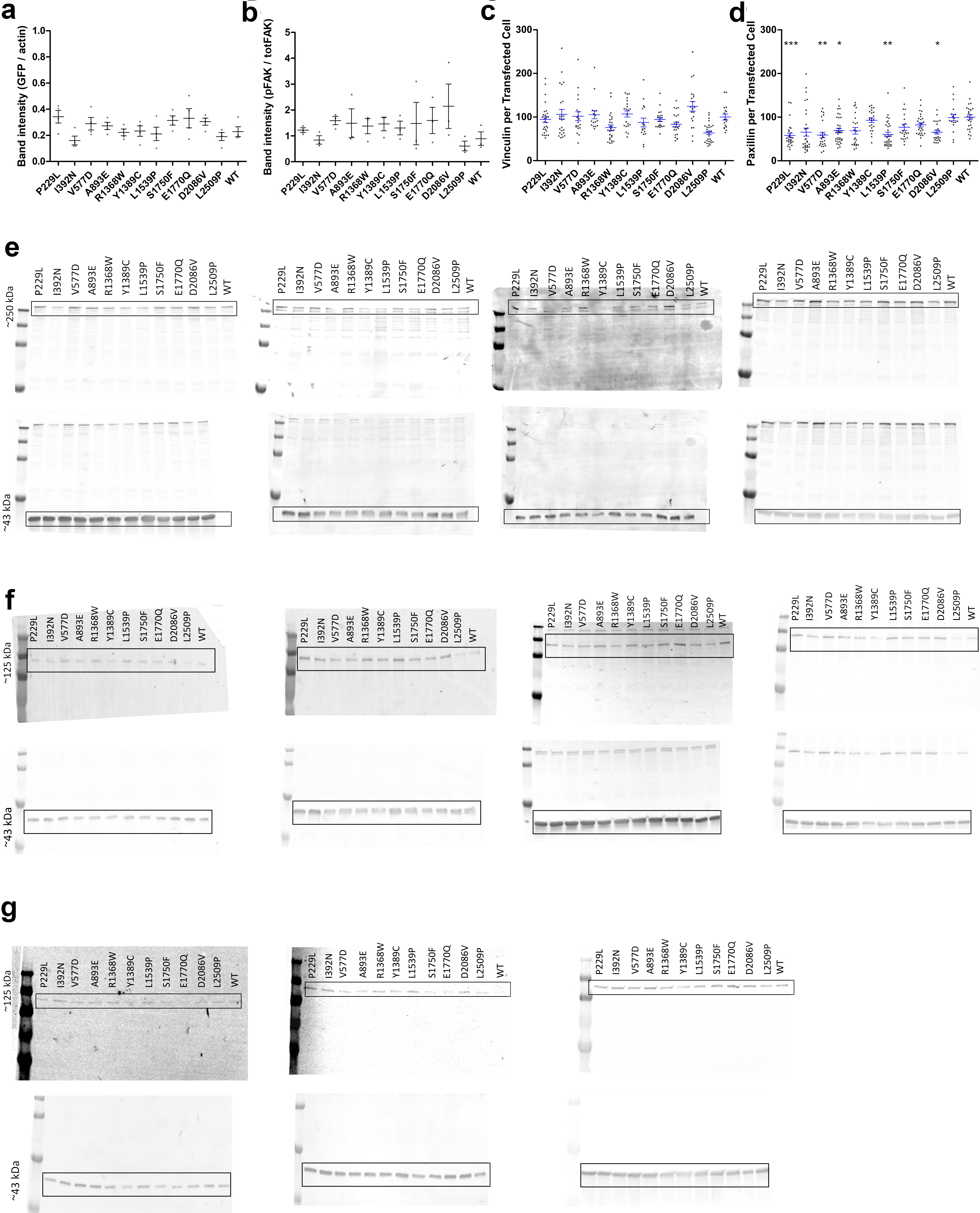

Supplement: Supplementary file 4 — Supplementary Information 4. [file 41598_2020_77911_MOESM4_ESM.tiff]

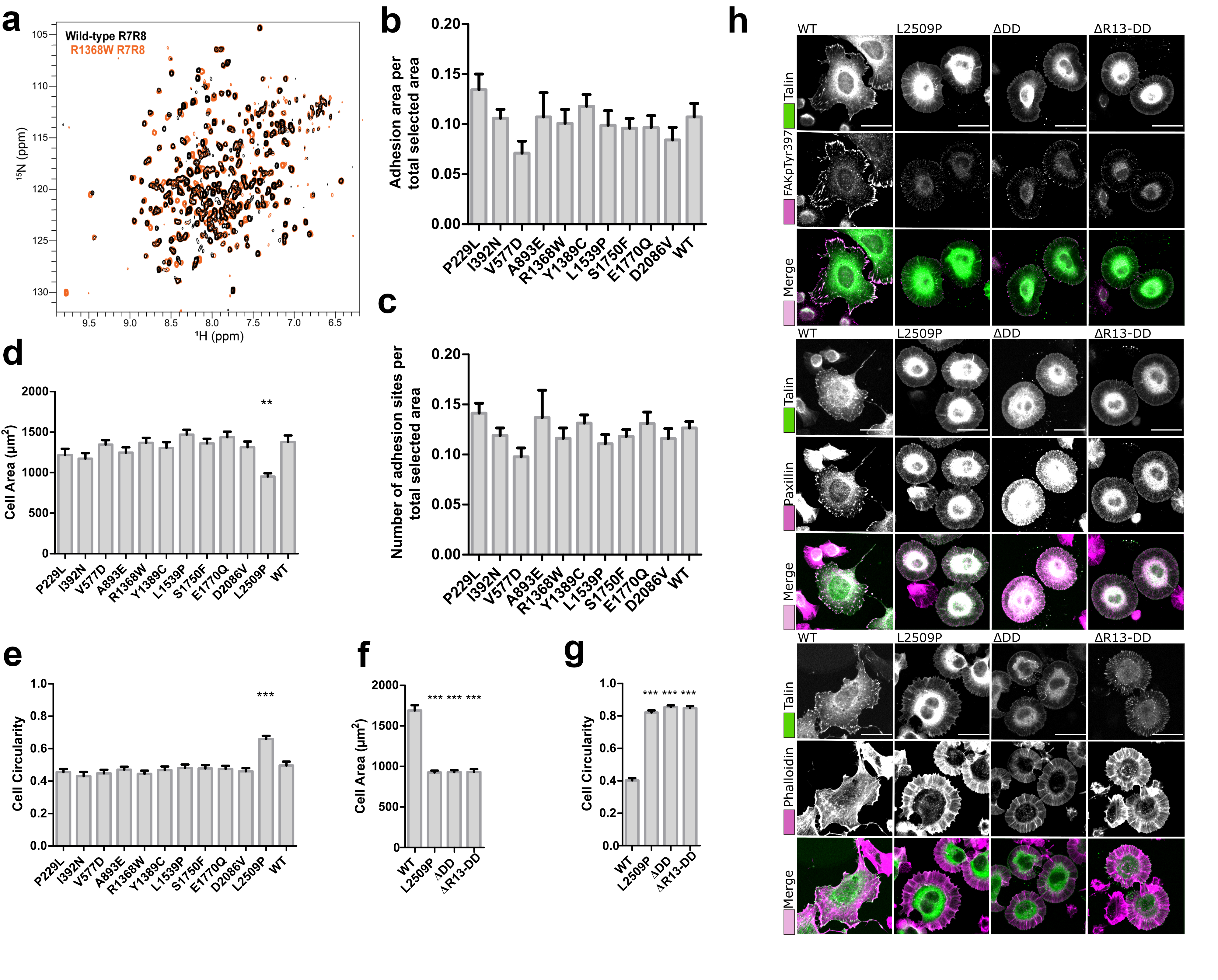

Supplement: Supplementary file 5 — Supplementary Information 5. [file 41598_2020_77911_MOESM5_ESM.tiff]

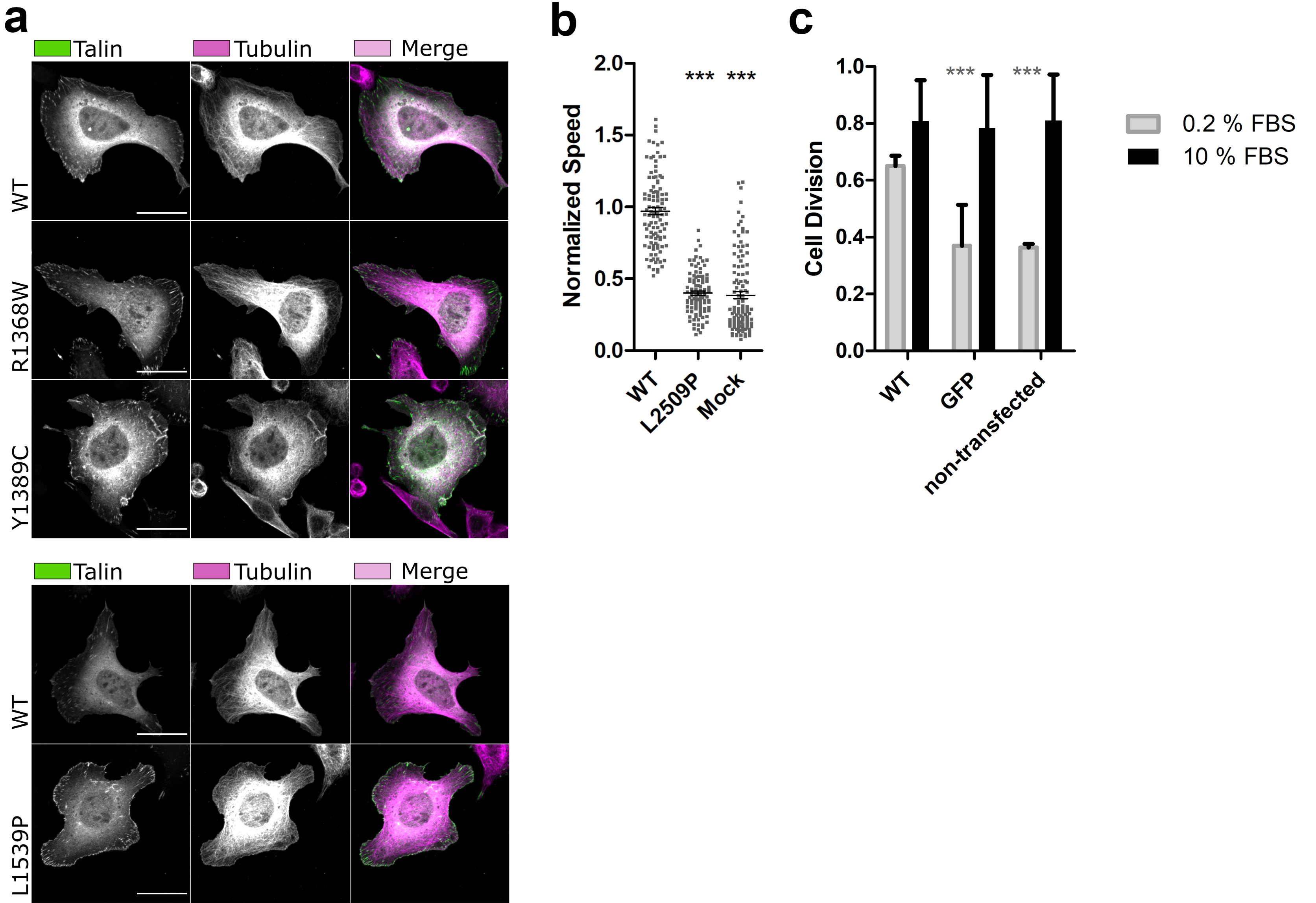

Supplement: Supplementary file 6 — Supplementary Information 6. [file 41598_2020_77911_MOESM6_ESM.tiff]

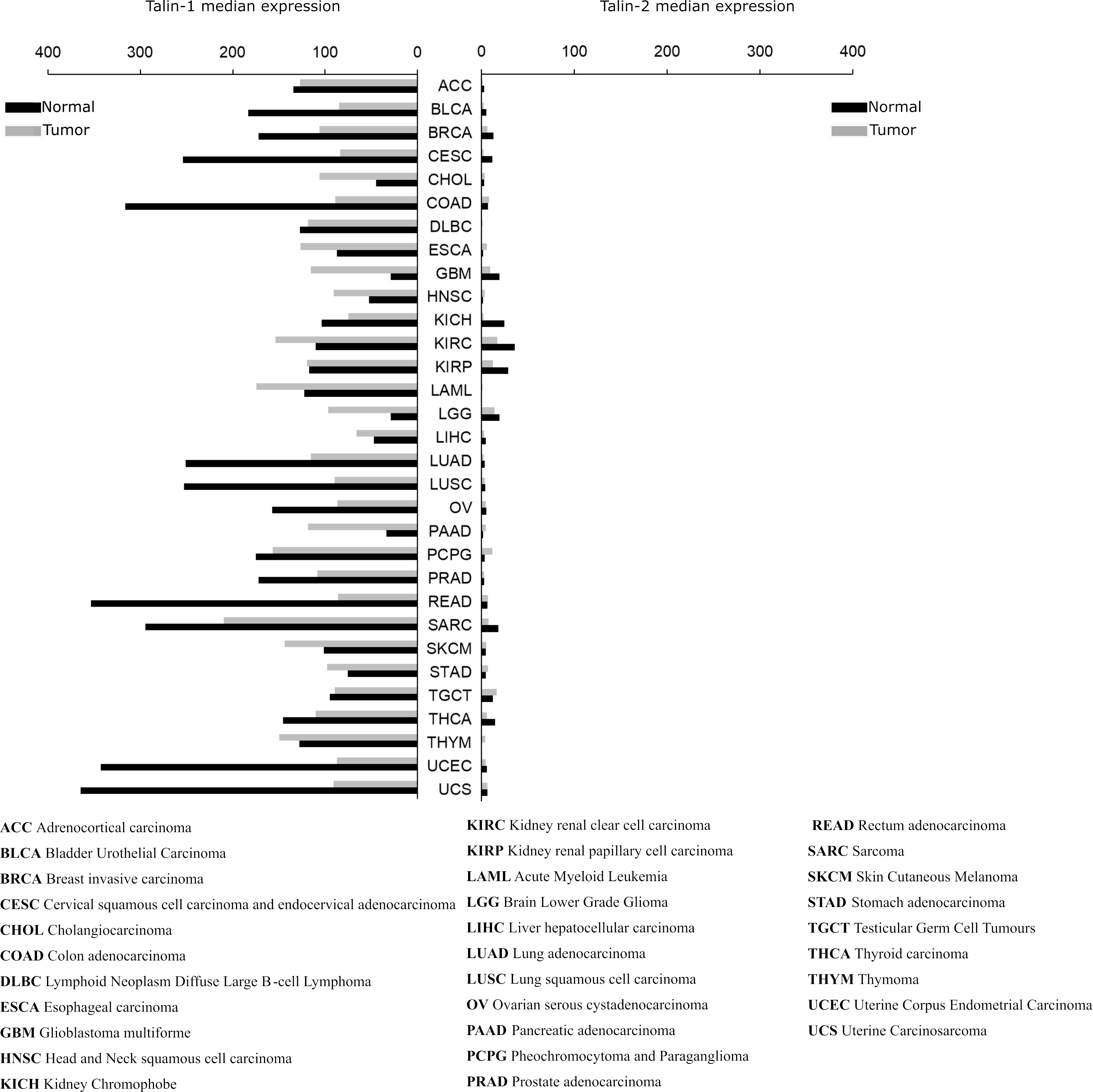

Supplement: Supplementary file 7 — Supplementary Information 7. [file 41598_2020_77911_MOESM7_ESM.tiff]
